# Correspondence

**Published:** 1907-11

**Authors:** 

**Affiliations:** New York


### CORRESPONDENCE

**Messrs. Fairchild Bros. & Foster, of New York, detect Chicago Druggists in Substituting. They Serve Notice that the Fight against Substitution will be Carried to a Finish.**

*Editor Buffalo Medical Journal:*

Sir: We received a request from the Ways and Means Committee of the National Association of Retail Druggists to subscribe to the fund for the entertainment of the members of the N. A. R. D. at its forthcoming Convention in the City of Chicago. In view of previous experience it occurred to us to take steps to ascertain to what extent that "community of interest" expressly implied and naturally anticipated, did actually prevail. We therefore caused prescriptions, in which Essence of Pepsine, Fairchild's was plainly specified, to be sent to the following mentioned Chicago drug stores: I. M. Light, 143 E. 35th St.; Herman Lieberman, 528 So. Halsted St.; J. S. Link, 649 West 21st St.; C. P. Girten, 7100 Harvard Ave.; H. J. Holthoefer, N. W. Cor. State and 32d Sts.; John J. Boehm, 748 So. Halsted St.; William W. Klore, 2354 State St.; William P. Knoche, N. E. Cor. 61st & Halsted Sts.; Moyen Bros., (Geo. F. W. Moyen), 1595 Milwaukee Ave. Cor. Armitage.

Each vial so dispensed was upon receipt immediately sealed in its original wrapper and delivered to us, with complete data in every detail from the writing to the dispensing and sealing of the prescription. Upon examination the fluid dispensed by each of these druggists was found to be a substitute—some preparation not made by us and not Fairchild's Essence.

These substitutes were unlike in composition; they were of varying degrees of inferiority *and some practically worthless* for the purpose for which Fairchild's Essence of Pepsine is esteemed and employed.

There is one significant fact that should also be mentioned, and that is that the price at which the substitutes were sold is that charged the patient by pharmacists who dispense Fairchild's Essence of Pepsine when it is ordered.

We therefore bring these facts to your attention, again stating our intention to fight against substitution and for a "square deal".

Very truly yours,

FAIRCHILD BROS. & FOSTER.

New York, September 12, 1907.

